# Carbon Monoxide Protects against Hepatic Ischemia/Reperfusion Injury via ROS-Dependent Akt Signaling and Inhibition of Glycogen Synthase Kinase 3****β****

**DOI:** 10.1155/2013/306421

**Published:** 2013-12-18

**Authors:** Hyo Jeong Kim, Yeonsoo Joe, Jin Sun Kong, Sun-Oh Jeong, Gyeong Jae Cho, Stefan W. Ryter, Hun Taeg Chung

**Affiliations:** ^1^School of Biological Sciences, University of Ulsan, Ulsan 680-749, Republic of Korea; ^2^Department of Anatomy, School of Medicine, and Institute of Health Sciences, Gyeongsang National University, Jinju 660-701, Republic of Korea; ^3^Department of Medicine, Pulmonary and Critical Care Medicine, Brigham and Women's Hospital, Harvard Medical School, Boston, MA 02115, USA

## Abstract

Carbon monoxide (CO) may exert important roles in physiological and pathophysiological states through the regulation of cellular signaling pathways. CO can protect organ tissues from ischemia/reperfusion (I/R) injury by modulating intracellular redox status and by inhibiting inflammatory, apoptotic, and proliferative responses. However, the cellular mechanisms underlying the protective effects of CO in organ I/R injury remain incompletely understood. In this study, a murine model of hepatic warm I/R injury was employed to assess the role of glycogen synthase kinase-3 (GSK3) and phosphatidylinositol 3-kinase (PI3K)-dependent signaling pathways in the protective effects of CO against inflammation and injury. Inhibition of GSK3 through the PI3K/Akt pathway played a crucial role in CO-mediated protection. CO treatment increased the phosphorylation of Akt and GSK3-beta (GSK3**β**) in the liver after I/R injury. Furthermore, administration of LY294002, an inhibitor of PI3K, compromised the protective effect of CO and decreased the level of phospho-GSK3**β** after I/R injury. These results suggest that CO protects against liver damage by maintaining GSK3**β** phosphorylation, which may be mediated by the PI3K/Akt signaling pathway. Our study provides additional support for the therapeutic potential of CO in organ injury and identifies GSK3**β** as a therapeutic target for CO in the amelioration of hepatic injury.

## 1. Introduction

Hepatic ischemia/reperfusion (I/R) injury is a cause of significant morbidity and mortality after liver transplantation, hemorrhagic shock, and extended liver resection for cancer. The pathophysiology of liver I/R injury includes both initial cellular damage due to ischemia as well as delayed liver dysfunction following reperfusion-initiated and inflammation-induced hepatocellular damage [1]. During I/R injury, Toll-like receptor 4 (TLR4) activation leads to neutrophil infiltration and may promote liver damage through the increase of proinflammatory cytokines. I/R injury can cause chronic inflammation and disease through TLR4 activation [2].

TLR4 activation by lipopolysaccharide (LPS) can be suppressed by the cytoprotective heme oxygenase-1/carbon monoxide (HO-1/CO) system [3]. CO, a reaction product of HO-1 activity, has been shown to have potent anti-inflammatory, antiproliferative, and antiapoptotic effects and thereby mimics the cytoprotective effects of HO-1 [4]. CO, when applied at low concentration, can confer anti-inflammatory effects in macrophages and protect endothelial cells and hepatocytes against cytotoxic agents [5, 6]. Likewise, the exogenous application of gaseous CO also protects against cold hepatic I/R injury in the *ex vivo* isolated liver perfusion model [7]. Although CO inhalation or pharmacological application using CO-releasing molecules (CORMs) has been reported to ameliorate I/R injury in various animal models [7–9], the molecular mechanisms underlying the cytoprotective effects of the HO-1/CO system on hepatic I/R injury have not been well studied.

Recent studies have shown that inhibition of glycogen synthase kinase 3*β* (GSK3*β*) by Ser9 phosphorylation can confer cardioprotective effects during myocardial infarction [10, 11], and ameliorate liver I/R injury [12]. GSK3*β* activity has recently been identified in a number of studies as crucial in the regulation of the inflammatory response. Phosphatidylinositol-3-kinase (PI3 K)/Akt-dependent inhibition of GSK3*β* activity in monocytes can regulate TLR-dependent activation [13, 14]. Although hepatic I/R injury has been reported to be ameliorated by inhibition of GSK3*β*, the molecular mechanisms by which GSK3*β* confers cytoprotective effects in hepatic I/R injury through TRL4 modulation have not been well studied.

In this study, we establish a signaling pathway by which CO can confer anti-inflammatory protection for liver homeostasis. We demonstrate that CO inhibits GSK3*β* activity through a PI3 K/Akt-mediated pathway, leading to the downregulation of TLR4-dependent proinflammatory cytokines, and the upregulation of IL-10. Our results further validate the use of CO as a pharmacological cytoprotective agent against hepatic I/R injury and identify GSK3*β* as a major therapeutic target of CO action in the liver.

## 2. Materials and Methods

### 2.1. Animal

Male C57BL/6 wild type (WT) mice at 8–10 weeks of age were purchased from the Orient Bio (Seoul, Korea). Animals were maintained in a specific pathogen-free facility. Animal studies were approved by the University of Ulsan Animal Care and Use Committee.

### 2.2. Cell Culture

The human hepatocarcinoma cell line (HepG2) and the murine macrophage cell line, RAW 264.7, were cultured in DMEM (Gibco, Grand Island, NY). All media was supplemented with 10% fetal bovine serum and a 100 units/mL penicillin-streptomycin mixture (Gibco).

### 2.3. Carbon Monoxide Treatment

To evaluate the protective effect of inhaled CO, animals were randomly assigned to receive preconditioning with room air or room air supplemented with 250 parts per million (ppm) CO, for 12 hours in a sealed exposure chamber prior to the experiment. Mice were exposed to CO 250 ppm for 1 hour and 6 hours after reperfusion.

### 2.4. Mouse Liver I/R Injury Model

We used a well-established mouse model of warm hepatic ischemia followed by reperfusion [15]. An atraumatic clip was used to interrupt the arterial/portal venous blood supply to the cephalad liver lobes. After 90 minutes the clip was removed; mice were sacrificed at various time points of reperfusion. Sham wild-type (WT) controls underwent the same procedure, but without vascular occlusion. Mice were exposed to compressed air or carbon monoxide (CO), at 250 parts per million (ppm). CO or room air was given to the mouse overnight prior to the liver ischemia and during reperfusion. In some experiments, the PI3 K inhibitor LY294002 (Sigma, St Louis, MO, 0.5 mg/kg, *i.p.*) or vehicle (10% DMSO in PBS* i.p.*) was given 30 min prior to the ischemic insult.

### 2.5. Hepatocellular Damage Assay

To detect serum alanine aminotransferase (sALT), serum was collected from peripheral blood. ALT activity, an indicator of hepatocellular injury, was measured using the EnzyChrom Alanine Transaminase Assay Kit (BioAssay System, Hayward, CA).

### 2.6. Liver Histology

For histopathological observations, portions of liver were fixed in 10% neutral-buffered formalin solution and then dehydrated in graded alcohol. The fixed tissue was embedded in paraffin and sliced into 4 *μ*m thick sections. Tissue sections were mounted on regular glass slides, deparaffinized in xylene, rehydrated in decreasing concentrations of ethanol, and stained with hematoxylin and eosin (H&E). Overall pathological changes, including immune cell infiltration and hepatic cell necrosis, were diagnosed according to previously described methods [16].

### 2.7. Immunohistochemistry

For the detection of p-GS (S641) and p-GSK3*β* (S9) by immunohistochemistry, the tyramide signal amplification (TSA) biotin system (Perkin-Elmer, Waltham, MA) was used according to the protocols recommended by the manufacturer. Briefly, after blocking, the sections were incubated first with either anti-p-GS (S641) or anti-p-GSK3*β* (S9) antibody (Cell Signaling Technologies, Danvers, MA). After overnight incubation, the sections were washed and then incubated with biotinylated anti-rabbit IgG antibody, next with streptavidin-horseradish peroxidase (HRP), and then with the biotinyl tyramide amplification reagent. Deposition of the biotin-tyramide on tissue sections was visualized with streptavidin-HRP and the substrate diaminobenzidine (DAB; Merck, Darmstadt, Germany); and then the sections were then counterstained with hematoxylin.

### 2.8. Immunoprecipitation

Preparation of nuclear extracts was carried out using the Nuclear/Cytosol Fractionation Kit (BioVision, Milpitas, CA). Proteins in the cell lysates were immunoprecipitated with anti-CBP antibodies for 3 h at 4°C, followed by incubation with Dynabeads protein G overnight at 4°C. Proteins in the immunoprecipitates were resolved using SDS-PAGE, followed by Western blotting with anti-phospho-NF-*κ*B p65 and anti-phospho-CREB antibodies (Santa Cruz Biotechnology, Santa Cruz, CA).

### 2.9. SDS-PAGE Analysis and Immunoblotting

Harvested liver tissues and cells were lysed with mammalian lysis buffer containing phosphatase and protease inhibitors. Equal amounts of cell lysates were measured with the BCA protein assay reagent (Pierce Biotechnology, Rockford, IL). Lysates were boiled in sample buffer containing *β*-mercaptoethanol for 5 min. Proteins were then subjected to SDS-PAGE and transferred to polyvinylidene difluoride membranes (GE healthcare, Piscataway, NJ). After blocking with 5% skim milk in PBS, membranes were incubated with appropriate dilutions of antibodies at 4°C overnight as follows: polyclonal rabbit anti-phospho glycogen synthase kinase (Ser9), rabbit anti-phospho glycogen synthase (Ser641), mouse anti-glycogen synthase kinase, rabbit anti-glycogen synthase, rabbit anti-phospho CREB (Ser133), rabbit anti-phospho Akt (Ser473), rabbit anti-HMGB1 (Cell Signaling Technology, Danvers, MA), rabbit anti-CBP, rabbit anti-phospho-NF-*κ*B-p65 (Ser276), and *β*-actin (Santa Cruz Biotechnology, Santa Cruz, CA) were used. Membranes were then washed with 0.05% PBS-Tween 20 and incubated with a 1/5000 dilution of HRP-conjugated secondary Abs at room temperature for 1 h. Immunoreactivity was detected using the ECL detection system (GE Healthcare, Piscataway, NJ). Films were exposed at multiple time points to ensure that the images were not saturated.

### 2.10. Real-Time and Semiquantitative RT-PCR

Total RNA was prepared using Trizol reagent (Invitrogen, Carlsbad, CA). Three microgram of total RNA was used to synthesize the first-strand cDNA by using oligo-dT primers (QIAGEN, CA) and M-MLV reverse transcriptase (Promega, Madison, WI) according to the manufacturer's instructions. The synthesized cDNA was subjected to the PCR-based amplification. Semiquantitative RT-PCR was performed using Taq polymerase (Solgent, Daejeon, Korea). Real-time PCR was performed using SYBR Green PCR Master Mix (Qiagen, Valencia, CA) on an ABI 7500 Fast Real-Time PCR System (Applied Biosystems, Grand Island NY). PCR primer pairs were as follows: TNF: 5′-AGA CCC TCA CAC TCA GAT CAT CTT C-3′, 5′-TTG CTA CGA CGT GGG CTA CA-3′, IL-6: 5′-CGA TGA TGC ACT TGC AGA AA-3′, 5′-TGG AAA TTG GGG TAG GAA GG-3′ and IL-10: 5′-CAG TAC AGC CGG GAA GAC AA-3′, 5′-CAG CTT CTC ACC CAG GGA AT-3′.

### 2.11. Myeloperoxidase Assay

Neutrophil sequestration in liver was quantified by measuring tissue MPO activity. Tissue samples for MPO analysis were frozen in liquid nitrogen immediately after removal from the animal and were thawed and homogenized and centrifuged to remove insoluble materials. MPO activities were measured using a mouse myeloperoxidase DuoSet ELISA kit (R&D Systems, Minneapolis, MN) according to the manufacturer's instruction. The supernatants were analyzed for MPO levels by sandwich ELISA. The levels of MPO in organ extracts were expressed as ng/mg of protein.

### 2.12. Statistical Analysis

All data were expressed as mean ± SD. Differences between experimental groups were compared using the Student's two-tailed unpaired *t*-test.

## 3. Results

### 3.1. Carbon Monoxide Inhalation Protects Liver Ischemia/Reperfusion Injury via AKT-GSK3*β* Activation in Mice

**Figure 1:**
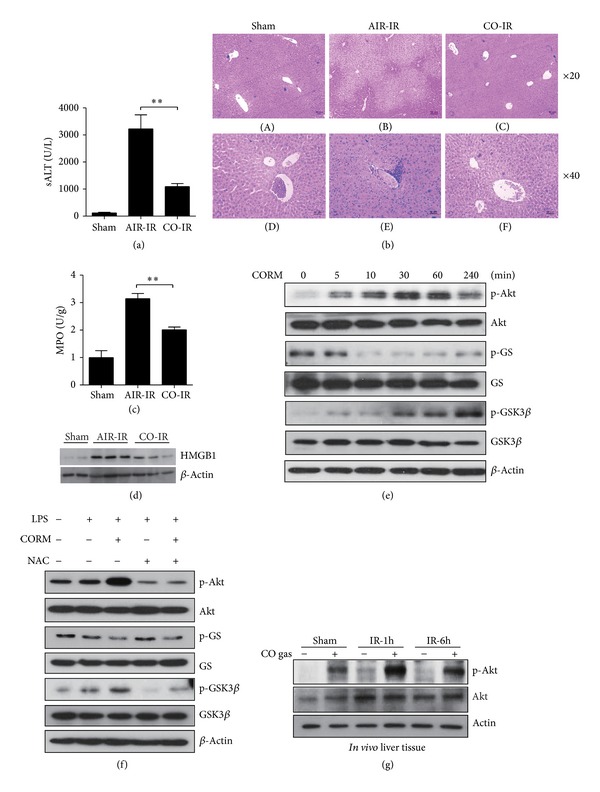
Pretreatment of mice with CO gas inhalation ameliorates liver I/R injury via AKT-GSK3*β* activation. Mice were subjected to 90 minutes liver warm ischemia, followed by 6 h reperfusion. (a) Hepatocellular function was evaluated by sALT (IU/L). (b) Representative liver histology of ischemic liver lobes. (c) Liver neutrophil accumulation, assessed by MPO activity. Data represent the mean ± standard deviation (SD) (*N* = 4–6 samples/group). **P < 0.01. (d) Hepatic HMGB1 expression in liver tissue was assessed by Western blot analysis at 1 h and 6 h of reperfusion. Total cell lysates were analyzed for HMGB1 and *β*-actin protein levels by Western blot analysis. (e) Western-blot analysis of phospho (p)-GSK3*β* (Ser 9), p-GS (Ser641), and p-Akt in HepG2 cells after treatment with CORM2 (50 *μ*M) at the indicated times. (f) RAW264.7 cells were stimulated with 10 ng/mL of LPS for 30 minutes in the absence or presence of CORM2 and the ROS scavenger, N-acetyl-cysteine (NAC). Total cell lysates were analyzed for phosphorylated GS, GSK3*β*, and Akt as well as total GS, GSK3*β*, Akt, and *β*-actin protein levels by Western immunoblot analysis. (g) Mice were subjected to 90 minutes of liver warm ischemia, followed by 1 h or 6 h reperfusion. Liver tissue was analyzed by Western blotting of p-Akt and total Akt. *β*-Actin served as the standard.

Carbon monoxide (CO) has been shown to exert protective effects in various tissue models of I/R injury [8, 9, 17]. We analyzed the effect of CO on hepatocellular function in mouse livers subjected to 90 min of warm ischemia followed by 6 h reperfusion. As shown in Figure 1(a), sALT levels in mice subjected to hepatic I/R were decreased in animals pretreated with CO gas, as compared with room air (3225 ± 891 U/L *versus * 1091 ± 230 U/L, respectively, *P* < 0.01). Figure 1(b) shows that hepatocellular necrosis (*panels *(A)–(C)) and immune cell infiltration (*panel* (D)–(F)) observed in the air-treated I/R group were markedly reduced with CO inhalation. Furthermore, myeloperoxidase (MPO) activity, reflecting liver neutrophil activity, was decreased in the CO inhalation group, compared with the air-treated group after hepatic I/R (5.371 ± 0.902 ng/mg *versus*  10.468 ± 1.700 ng/mg, resp., *P* < 0.01) (Figure 1(c)). Since HMGB1, an inflammatory cytokine, can promote liver damage following I/R injury [18, 19], we examined HMGB1 expression in our hepatic I/R model. As shown in Figure 1(d), I/R caused an increase in hepatic HMGB1 expression when compared with sham control mice. CO inhalation markedly attenuated the expression of HMGB1 during I/R injury compared to air-treated mice subjected to I/R (Figure 1(d)).

Previous reports have shown that HO-1 derived CO can activate the PI3 K-Akt pathway [20] and thereby confer protection against cardiac I/R injury [21]. To investigate whether CO activates Akt signaling in hepatic cell lines, we assessed the expression of phosphorylated (p)-Akt and of GSK3*β*, which is negatively regulated by Akt. As shown in Figure 1(e), CO increased the phosphorylation of both Akt and GSK3*β* in HepG2 cells. Conversely, CO decreased the phosphorylation of GS, the substrate of GSK3*β*.

A previous study has shown that both endogenous and exogenous CO can increase cellular ROS generation [22]. ROS can exert a critical role in maintaining homeostasis by protecting the host against excessive inflammatory responses [23]. Because activation of the phosphatidylinositol 3-kinase (PI3 K)/Akt pathway by ROS signaling can function in cellular adaptation [24, 25], we sought to determine whether CO-mediated ROS generation can increase LPS-induced phosphorylation of Akt and GSK3. As shown in Figure 1(f), we used the ROS scavenger N-acetyl-cysteine (NAC) to determine whether phosphorylation of Akt and GSK3*β* was modulated by ROS generation. Treatment with CO dramatically increased LPS-induced phosphorylation of Akt and GSK3*β*. Inhibition of ROS generation with NAC attenuated LPS/CO-induced phosphorylation of Akt and GSK3*β*. These results suggest that CO stimulates LPS-dependent PI3 K/Akt-GSK3*β* phosphorylation *via* enhanced ROS production.

Therefore, to examine whether CO activates PI3 K/Akt signaling during hepatic I/R injury, we examined the expression of p-Akt in mice subjected to hepatic I/R. There was a significant increase in p-Akt in the CO-treated mice subjected to hepatic I/R injury at 1 hour of reperfusion relative to air-treated controls, which was sustained until 6 hours of reperfusion (Figure 1(g)). These findings suggested that the Akt-GSK3*β* axis may be involved in CO-mediated protection in the liver I/R model.

### 3.2. The Protective Effect of CO in Liver Ischemia/Reperfusion Injury Is Dependent on the Inactivation of GSK3 Activity

**Figure 2:**
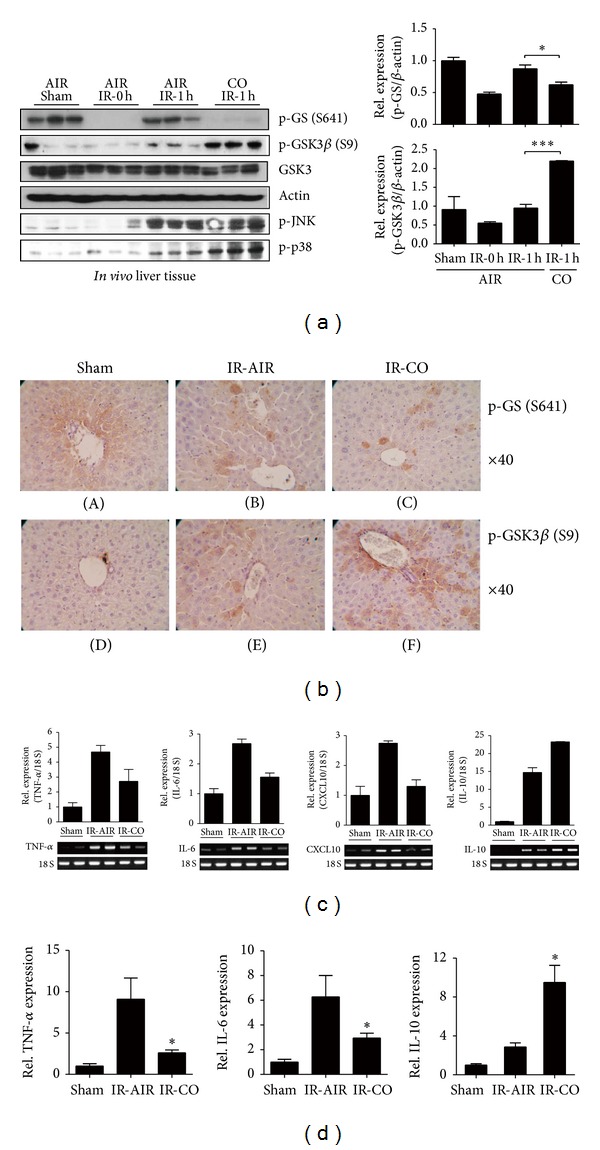
Inhibition of GSK3*β* by CO inhalation ameliorates liver I/R injury. Mice were sham-operated or subjected to 90 minutes hepatic warm ischemia followed by 1 hour reperfusion. Recipients were treated with air or CO gas (250 ppm) inhalation. (a) Liver samples, harvested 1 hour later, were subjected to Western blot analysis of phospho (p)-GS (GS 641), p-GSK3*β* (S9), p-JNK, and p-p38. *β*-Actin was used as an internal control. *P < 0.05, ***P < 0.001. (b) Immunohistochemical staining of GSK3 inhibition. Mice were sacrificed, liver tissues were harvested, and the tissue slices were processed for formalin-fixed paraffin embedding. Hematoxylin counterstaining after 3-amino-9-ethylcarbazole-based immunohistochemical staining was used to detect GS 641 ((A)–(C), red/brown) and phosphorylation of GSK3*β* S9 ((D)–(F), red/brown). ((c) and (d)) Quantitative RT-PCR-assisted detection of TNF-*α*, IL-6, CXCL10, and IL-10 gene expression at 1 hour or 6 hours in liver tissue. Data were normalized to 18S gene expression. Data shown represent the mean ± S.D. (*N* = 4-5/group), *P < 0.05.

I/R stimulation has been previously shown to trigger GSK3*β* phosphorylation in mouse liver as a self-regulatory mechanism [12]. Given that inhibition of GSK3*β* is involved in amelioration of liver pathology, we hypothesized that GSK3*β* inhibition may represent the underlying mechanism for CO-mediated liver protection. To evaluate the potential role of GSK3*β* in CO-mediated liver protection, we analyzed the effects of CO on GSK3*β* phosphorylation at Ser-9 and glycogen synthase (GS) phosphorylation at Ser-641, the substrate of GSK3*β*. As shown in Figure 2(a), mice exposed to CO inhalation displayed marked increases in the phosphorylation of GSK3*β* in the ischemic livers, compared with the sham control and air-inhaled groups. Conversely, the level of GS phosphorylation was decreased in ischemic mice given CO inhalation, compared with the air inhalation group. According to a previous report (26), the decrease of GS phosphorylation that occurs in response to I/R stimulation alone is due to GS phosphatase activation by I/R. Thus, transient ischemia induces GS activation and glycogen synthesis *in vivo*. These observations suggest that this occurs through a different mechanism than through the increase of pGSK3-*β*, as observed with CO treatment.

Previous studies have demonstrated that the cytoprotective effects of CO involve p38 MAPK signaling [7]. Consistently, we found that phosphorylated p38 MAPK was increased by CO inhalation in the livers of mice subjected to hepatic I/R, compared with air-treated control mice (Figure 2(a)). However, JNK phosphorylation was not altered by CO.

We used immunohistochemistry to examine whether CO-mediated liver protection was associated with GSK3 inhibition. As shown in Figure 2(b), inhalation with CO gas increased GSK3*β* phosphorylation in ischemic liver and reduced GS phosphorylation, as compared with the air-inhalation group. Consistent with immunohistochemistry data, decreased mRNA levels of TNF-*α*, IL-6, and CXCL10 were consistently found in the livers of mice subjected to CO-inhalation, compared with those of the air-inhalation group (Figure 2(c)). TNF-*α* and IL-6 proteins production was also inhibited by CO (Figure 2(d)). The anti-inflammatory cytokine IL-10 was significantly increased by CO inhalation in mice subjected to hepatic I/R injury (Figures 2(c) and 2(d)).

### 3.3. CO Dependent Inhibition of GSK3 and Attenuation of Liver Injury Involve PI3 K Signaling in Mice

**Figure 3:**
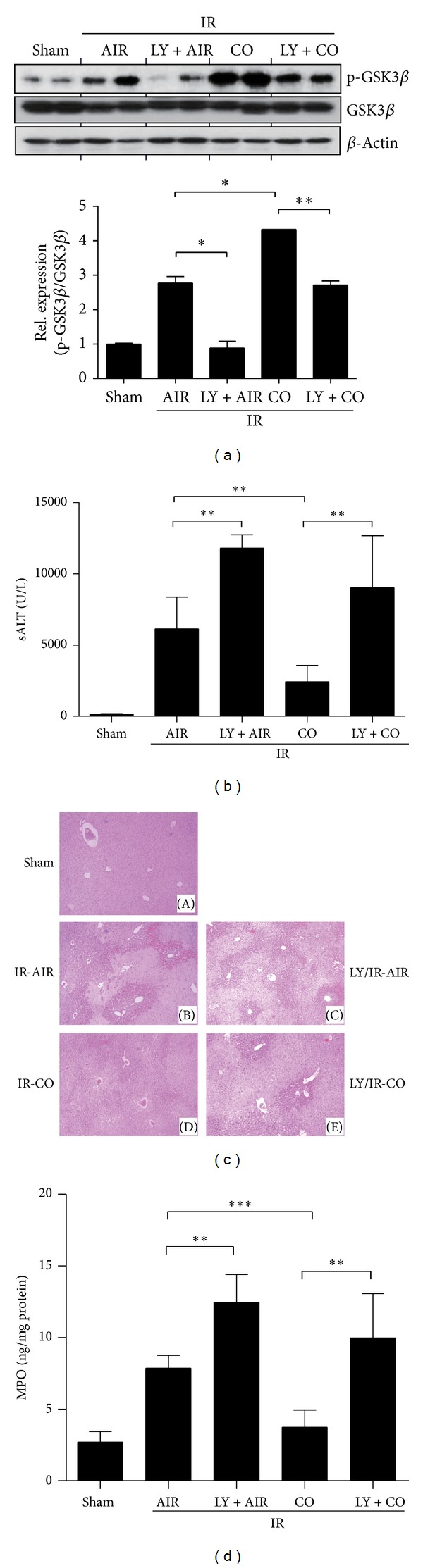
PI3 K blockade restores liver I/R injury in mice pretreated with CO inhalation. Mice were treated with CO gas (CO), LY294002 (LY), or both or vehicle at 30 minutes prior to the liver ischemia insult, as described in Materials and Methods. Liver samples were harvested at 6 hours after-reperfusion. (a) Proteins were analyzed by Western blotting with Abs against phosphorylated or total GSK3*β* and *β*-actin. Sixty minutes ischemia time was used to show the effect of PI3 K inhibition in liver I/R injury. *P < 0.05, **P < 0.01. (b) Average sALT levels in different experimental groups were measured. sALT levels were measured at 6 hours of reperfusion. (c) Representative liver histology (H&E staining) is shown. To establish the functional relationship between PI3 K and GSK3*β*, CO inhalation was administered 12 hr and LY 30 minutes prior to the ischemic insult and 6 hr after reperfusion. (d) Liver neutrophil accumulation, assessed by MPO activity. Data represent mean ± S.D. (*N* = 4–6 samples/group). **P < 0.01, ***P < 0.001.

The PI3 K-Akt pathway has been shown *in vitro* to regulate GSK3*β* phosphorylation through the activation of TLR4 [26]. We used LY294002, an irreversible PI3 K-specific inhibitor, to address the functional role of PI3 K/Akt signaling in CO-mediated liver cytoprotection. Indeed, LY294002-treated mice displayed significantly lower levels of phosphorylated GSK3*β* in the liver after I/R (Figure 3(a)). Increased sALT levels were consistently found in mice treated with the PI3 K inhibitor with or without CO inhalation (Figure 3(b), 11791 ± 940 U/L and 9012 ± 3657 U/L, resp.), compared with CO inhalation alone (2423 ± 1145 U/L, *P* < 0.01) (Figure 3(b)). Unlike mice pretreated with CO, which displayed minimal liver damage (Figure 3(c), *panel *(D)), mice given the PI3 K inhibitor revealed significant hepatocellular necrosis, cytoplasmic vacuolization, and sinusoidal congestion (*panel *(C)). Livers of animals treated with the PI3 K inhibitor after CO inhalation showed moderate to severe hepatocellular changes (*panel *(E)). As shown in Figure 3(d), MPO levels were elevated in PI3 K inhibitor-treated mice (12.458 ± 1.947 ng/mg), compared with DMSO controls (7.864 ± 0.891 ng/mg, *P* < 0.01). In contrast, livers from the CO inhalation group showed decreased MPO activity (3.738 ± 1.203 ng/mg), compared with the group subjected to PI3 K inhibitor treatment after CO inhalation (9.964 ± 3.099, *P* < 0.01). Thus, PI3 K/Akt-dependent GSK3*β* phosphorylation, a therapeutic target of CO, serves as a self-regulatory mechanism of liver homeostasis to limit the excessive I/R-induced tissue damage.

### 3.4. CO-Mediated GSK3 Inhibition via PI3 K/AKT Signaling Regulates the LPS-Mediated Inflammatory Response *In Vitro*

**Figure 4:**
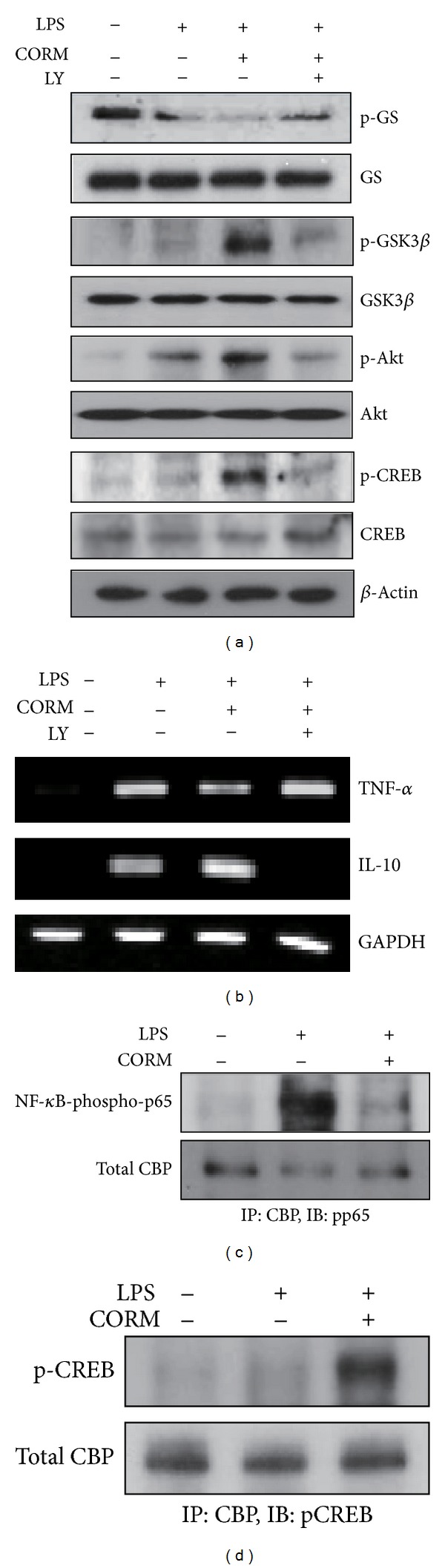
CO-induced PI3 K/Akt-dependent GSK3 inactivation regulates TLR4 responses and affects the ability of CREB and NF-*κ*B-p65 to associate with CBP. RAW264.7 cells were stimulated with 100 ng/mL of LPS for 1 hour in the absence or presence of CORM2 and the PI3 K inhibitor (LY294002). (a) Total cell lysates were analyzed for phosphorylated GS, GSK3*β*, Akt, and CREB as well as total GS, GSK3*β*, Akt, CREB, and *β*-actin protein levels by Western immunoblot analysis. (b) The mRNA expression of TNF-*α* and IL-10 was determined by semiquantitative RT-PCR. GAPDH was used as an internal control. ((c) and (d)) RAW264.7 cells were stimulated with 0.1 *μ*g/mL of LPS for 1 hour in the absence or presence of CORM2 (50 *μ*M) and nuclear extracts were obtained. Interaction of CBP with NF-*κ*B p65 and CREB was assessed by immunoprecipitation of CBP followed by immunoblotting for NF-*κ*B or CREB. Total CBP served as the input standard.

Previous reports have shown that LPS stimulation in monocytes can result in Ser9 phosphorylation of GSK3*β* in a PI3 K/Akt-dependent pathway [27] and that this pathway downregulates TLRs-dependent inflammatory responses. To investigate the cellular mechanisms underlying our *in vivo* findings, we analyzed whether CO-dependent activation of the PI3 K/Akt pathway may regulate TLR4-dependent inflammatory responses through GSK3*β* inhibition *in vitro*. LPS-stimulated macrophages were first pretreated with the PI3 K inhibitor (LY294002) and then treated with a CO releasing molecule (CORM2). Western blot analysis showed that treatment with CORM2 dramatically increased LPS-induced phosphorylation of Akt, GSK3*β*, and its downstream target, the cAMP response element-binding protein (CREB). However, pretreatment with PI3 K inhibitor (LY294002) inhibited phosphorylation of GSK3*β*, Akt, and CREB in LPS-stimulated macrophages treated with CORM2 (Figure 4(a)). In addition, GSK3*β* inactivation by CORM2 significantly reduced TNF-*α* and increased IL-10 gene expression in response to LPS (Figure 4(b)). The modulatory effects of CORM2 on TNF-*α* and IL-10 expression were inhibited by LY294002. These results demonstrate that CO-induced GSK3*β* inactivation is mediated by the PI3 K/Akt signaling pathway and consequently modulates the TLR4-driven inflammatory response.

### 3.5. Negative Regulation of GSK3 Activity by CO Affects the Associations of NF-*κ*B p65 and CREB with CBP That Regulates the Production of IL-10

We next sought to validate the cellular mechanism by which CORM2-induced phosphorylation of GSK3*β* leads to the upregulation of the anti-inflammatory cytokine IL-10. Previous studies have identified CREB, a target of GSK3*β*, as an important transcription factor regulating IL-10 production in monocytes [28]. GSK3*β* can negatively regulate the activation and DNA-binding activity of CREB [28]. GSK3*β* increases the binding of NF-*κ*B p65 to the coactivator CREB-binding protein (CBP), leading to proinflammatory gene activation, which competes against the binding of CREB to CBP, the latter regulating IL-10 expression [27].

We sought to determine whether GSK3*β* inactivation by CORM2 influenced the ability of CREB and NF-*κ*B p65 to associate with CBP. As shown in Figures 4(a) and 4(b), LPS-stimulated RAW 264.7 cells had increased association of p65 with CBP relative to unstimulated control cells. However, LPS-stimulated cells that were pretreated with CORM2 showed a considerable decrease in the association of p65 with CBP (Figure 4(c)), whereas the binding of CREB to CBP was potently augmented (Figure 4(d)), consistent with GSK3*β* inhibition.

## 4. Discussion

In the current study, we identify the GSK3*β* pathway as a novel therapeutic target of the anti-inflammatory effects of CO in a hepatic I/R injury model. The GSK3*β* pathway has previously been identified as an important target of inflammatory regulation. GSK3*β* is a proline-directed Ser/Thr kinase that phosphorylates a number of substrates including glycogen synthase (GS) as well as constituents of numerous intracellular signaling pathways including SMAD3, *β*-catenin, NOTCH2, CREB, and others [29–31]. Recent studies show that downregulation of GSK3*β* can negatively regulate the inflammatory response and protect mice from endotoxin shock [26]. In this model, GSK3*β* inhibition was associated with increased cAMP-response element binding (CREB) protein DNA binding activity, resulting in the increased production of the anti-inflammatory cytokine IL-10 and the decreased NF-*κ*B-dependent production of pro-inflammatory genes [26]. Recent studies have shown that inhibition of GSK3*β* ameliorates liver I/R injury through an IL-10 mediated immune regulatory mechanism [12]. During myocardial infarction, GSK3 inhibition with pharmacological inhibitors has been shown to exert a cardioprotective effect, potentially related to inhibition of mitochondrial permeability transition pore opening [10].

Carbon monoxide (CO), which can be applied by inhalation or by pharmacological delivery with CORMs, continues to show promise as an anti-inflammatory therapeutics in several models of organ I/R injury. For example, inhaled CO conferred tissue protection in rodents subjected to lung I/R injury, as evidenced by reduced markers of apoptosis, which depended on activation of the MKK3/p38 MAPK pathway [8, 9]. Additional mechanisms for CO-mediated protection during lung I/R include the derepression of the fibrinolytic axis and downregulation of the proinflammatory factor Egr-1 [32]. Furthermore, several studies demonstrated that pretreatment with CO donor compounds can ameliorate lung transplant-associated I/R injury with increased hepatic HSP70 expression [33] and can cause suppression of inflammatory responses *via* downregulation of the MEK/ERK1/2 signaling pathway [7].

In rodent models, CO has been shown to protect against acute liver injury caused by TNF*α* challenge [6] and can confer anti-inflammatory protection in hepatic I/R injury models [7, 17, 34]. CO preserved hepatic function *ex vivo* in an isolated perfused liver model subjected to cold ischemia injury, in part by upregulating the p38 MAPK pathway [34]. CO has been shown to protect against I/R injury during orthotropic rat liver transplantation by downregulating pro-inflammatory mediators, including TNF*α* and iNOS expression [17]. Furthermore, the protection afforded by CO in this model was also associated with the modulation of STAT1/STAT3 and inhibition of the MEK/ERK1/2 signaling pathway [7].

ROS have been shown to be involved in the counter-regulation of inflammation in response to LPS treatment through modulation of macrophage production of IL-10 [23]. Additionally, CO can act *via* inhibition of cytochrome *c* oxidase leading to the generation of low levels of reactive oxygen species (ROS) that mediate adaptive signaling pathways [35]. Our data demonstrate that CO inactivates GSK3*β* through a mechanism that involves increased ROS-induced Akt phosphorylation. These results provide evidence that the protective effects of CO are mediated through redox mechanisms that can lead to the activation of adaptive pathways.

The phosphatidylinositol-3-kinase (PI3 K)/Akt pathway represents another multifunctional signaling pathway that promotes cell survival under adverse conditions. Previous studies have implicated the PI3 K/Akt pathway in the cytoprotective effects of CO. For example, during anoxia/reoxygenation of pulmonary endothelial cells, CO treatment protected against apoptosis by upregulating the p38 MAPK and PI3 K/Akt-dependent STAT3 pathway. The protection afforded by CO in a cardiac I/R injury model *in vivo* has also been shown to be dependent on the activation of the p38 MAPK and PI3 K/Akt pathways.

The PI3 K/Akt pathway has been identified as an important regulator of GSK3 signaling. PI3 K/Akt-dependent inhibition of GSK3*β* activity in monocytes regulates TLR-dependent activation of inflammatory responses [13, 14]. Activation of the PI3 K/Akt pathway resulting in Ser9 dependent phosphorylation of GSK3*β* signaling pathway and inhibition of NF-*κ*B nuclear translocation were shown to contribute to cardioprotection during myocardial I/R injury [11]. Consistent with these observations, our *in vivo* data demonstrate that CO activates PI3 K/Akt signaling to promote GSK3*β* inhibition through phosphorylation at Ser9 during hepatic I/R injury. Furthermore, we have shown that activation of this pathway by CO therapy is a crucial mediator of the protection afforded by CO against hepatic injury and inflammation during I/R injury in mice.

GSK3*β* can differentially regulate TLR4-dependent signaling leading to modulation of anti-/proinflammatory cytokine balance. LPS stimulation in monocytes has been shown to result in Ser9 phosphorylation of GSK3*β* in a PI3 K/Akt-dependent pathway [26], which downregulates TLRs-dependent inflammatory responses. Hepatic warm ischemia and reperfusion (I/R) injury and inflammation are largely TLR4-dependent, whereas TLR4 appears to have marginal role in the early liver inflammatory response [36]. GSK3*β* inhibition was associated with hepatoprotection through the augmentation of the expression of the anti-inflammatory cytokine IL-10. Consistently, our results demonstrate that GSK3*β* inactivation by CO results in IL-10 upregulation in macrophages through a mechanism involving the augmentation of the binding of CREB to the nuclear co-activator CBP, leading and the suppression of the binding of NF-*κ*B p65 to the nuclear coactivator CBP.

In summary, our results establish a signaling pathway by which CO can confer anti-inflammatory protection in the liver: CO activates PI3 K/Akt which results in inhibition of GSK3*β* through Ser9 phosphorylation, leading to the downregulation of TLR4-dependent proinflammatory cytokines, and the upregulation of IL-10. The latter effect is mediated through activation of CREB and disruption of the p65/CBP interaction. Our results further validate the use of CO as a pharmacological cytoprotective agent against hepatic I/R injury and identify GSK3*β* as a major therapeutic target of CO action in the liver.

## References

[1] Jaeschke H (2003). Molecular mechanisms of hepatic ischemia-reperfusion injury and preconditioning. *The American Journal of Physiology: Gastrointestinal and Liver Physiology*.

[2] Lawrence T, Gilroy DW (2007). Chronic inflammation: a failure of resolution?. *International Journal of Experimental Pathology*.

[3] Wang XM, Kim HP, Nakahira K, Ryter SW, Choi AMK (2009). The heme oxygenase-1/carbon monoxide pathway suppresses TLR4 signaling by regulating the interaction of TLR4 with caveolin-1. *The Journal of Immunology*.

[4] Maines MD (1997). The heme oxygenase system: a regulator of second messenger gases. *Annual Review of Pharmacology and Toxicology*.

[5] Brouard S, Otterbein LE, Anrather J (2000). Carbon monoxide generated by heme oxygenase 1 suppresses endothelial cell apoptosis. *The Journal of Experimental Medicine*.

[6] Zuckerbraun BS, Billiar TR, Otterbein SL (2003). Carbon monoxide protects against liver failure through nitric oxide–induced heme oxygenase 1. *The Journal of Experimental Medicine*.

[7] Kaizu T, Ikeda A, Nakao A (2007). Protection of transplant-induced hepatic ischemia/reperfusion injury with carbon monoxide via MEK/ERK1/2 pathway downregulation. *The American Journal of Physiology: Gastrointestinal and Liver Physiology*.

[8] Zhang X, Shan P, Alam J, Davis RJ, Flavell RA, Lee PJ (2003). Carbon monoxide modulates Fas/Fas ligand, caspases, and Bcl-2 family proteins via the p38*α* mitogen-activated protein kinase pathway during ischemia-reperfusion lung injury. *The Journal of Biological Chemistry*.

[9] Zhang X, Shan P, Otterbein LE (2003). Carbon monoxide inhibition of apoptosis during ischemia-reperfusion lung injury is dependent on the p38 mitogen-activated protein kinase pathway and involves caspase 3. *The Journal of Biological Chemistry*.

[10] Gomez L, Paillard M, Thibault H, Derumeaux G, Ovize M (2008). Inhibition of GSK3*β* by postconditioning is required to prevent opening of the mitochondrial permeability transition pore during reperfusion. *Circulation*.

[11] Ha T, Hu Y, Liu L (2010). TLR2 ligands induce cardioprotection against ischaemia/reperfusion injury through a PI3K/Akt-dependent mechanism. *Cardiovascular Research*.

[12] Ren F, Duan Z, Cheng Q (2011). Inhibition of glycogen synthase kinase 3 beta ameliorates liver ischemia reperfusion injury by way of an interleukin-10-mediated immune regulatory mechanism. *Hepatology*.

[13] Fukao T, Yamada T, Tanabe M (2002). Selective loss of gastrointestinal mast cells and impaired immunity in P13K-deficient mice. *Nature Immunology*.

[14] Martin M, Schifferle RE, Cuesta N, Vogel SN, Katz J, Michalek SM (2003). Role of the phosphatidylinositol 3 kinase-Akt pathway in the regulation of IL-10 and IL-12 by *Porphyromonas gingivalis* lipopolysaccharide. *The Journal of Immunology*.

[15] Shen XD, Ke B, Zhai Y (2002). CD154-CD40 T-cell costimulation pathway is required in the mechanism of hepatic ischemia/reperfusion injury, and its blockade facilitates and depends on heme oxygenase-1 mediated cytoprotection. *Transplantation*.

[16] Tsai C-C, Huang W-C, Chen C-L (2011). Glycogen synthase kinase-3 facilitates con a-induced IFN-*γ*–mediated immune hepatic injury. *The Journal of Immunology*.

[17] Kaizu T, Nakao A, Tsung A (2005). Carbon monoxide inhalation ameliorates cold ischemia/reperfusion injury after rat liver transplantation. *Surgery*.

[18] Liu A, Dirsch O, Fang H (2011). HMGB1 in ischemic and non-ischemic liver after selective warm ischemia/reperfusion in rat. *Histochemistry and Cell Biology*.

[19] Tsung A, Sahai R, Tanaka H (2005). The nuclear factor HMGB1 mediates hepatic injury after murine liver ischemia-reperfusion. *The Journal of Experimental Medicine*.

[20] Zhang X, Shan P, Alam J, Fu X-Y, Lee PJ (2005). Carbon monoxide differentially modulates STAT1 and STAT3 and inhibits apoptosis via a phosphatidylinositol 3-kinase/Akt and p38 kinase-dependent STAT3 pathway during anoxia-reoxygenation injury. *The Journal of Biological Chemistry*.

[21] Fujimoto H, Ohno M, Ayabe S (2004). Carbon monoxide protects against cardiac ischemia—reperfusion injury in vivo via MAPK and Akt—eNOS pathways. *Arteriosclerosis, Thrombosis, and Vascular Biology*.

[22] Bilban M, Bach FH, Otterbein SL (2006). Carbon monoxide orchestrates a protective response through PPAR*γ*. *Immunity*.

[23] Deng J, Wang X, Qian F (2012). Protective role of reactive oxygen species in endotoxin-induced lung inflammation through modulation of IL-10 expression. *The Journal of Immunology*.

[24] Crack PJ, Taylor JM (2005). Reactive oxygen species and the modulation of stroke. *Free Radical Biology and Medicine*.

[25] Kandel ES, Hay N (1999). The regulation and activities of the multifunctional serine/threonine kinase Akt/PKB. *Experimental Cell Research*.

[26] McNulty PH, Luba MC (1995). Transient ischemia induces regional myocardial glycogen synthase activation and glycogen synthesis in vivo. *The American Journal of Physiology: Heart and Circulatory Physiology*.

[27] Martin M, Rehani K, Jope RS, Michalek SM (2005). Toll-like receptor–mediated cytokine production is differentially regulated by glycogen synthase kinase 3. *Nature Immunology*.

[28] Platzer C, Fritsch E, Elsner T, Lehmann MH, Volk HD, Prosch S (1999). Cyclic adenosine monophosphate-responsive elements are involved in the transcriptional activation of the human IL-10 gene in monocytic cells. *European Journal of Immunology*.

[29] Grimes CA, Jope RS (2001). CREB DNA binding activity is inhibited by glycogen synthase kinase-3*β* and facilitated by lithium. *The Journal of Neurochemistry*.

[30] Espinosa L, Inglés-Esteve J, Aguilera C, Bigas A (2003). Phosphorylation by glycogen synthase kinase-3*β* down-regulates Notch activity, a link for Notch and Wnt pathways. *The Journal of Biological Chemistry*.

[31] Furuhashi M, Yagi K, Yamamoto H (2001). Axin facilitates Smad3 activation in the transforming growth factor *β* signaling pathway. *Molecular and Cellular Biology*.

[32] Mishra S, Fujita T, Lama VN (2006). Carbon monoxide rescues ischemic lungs by interrupting MAPK-driven expression of early growth response 1 gene and its downstream target genes. *Proceedings of the National Academy of Sciences of the United States of America*.

[33] Lee L-Y, Kaizu T, Toyokawa H (2011). Carbon monoxide induces hypothermia tolerance in Kupffer cells and attenuates liver ischemia/reperfusion injury in rats. *Liver Transplantation*.

[34] Amersi F, Shen X-D, Anselmo D (2002). Ex vivo exposure to carbon monoxide prevents hepatic ischemia/reperfusion injury through p38 MAP kinase pathway. *Hepatology*.

[35] Zuckerbraun BS, Chin BY, Bilban M (2007). Carbon monoxide signals via inhibition of cytochrome c oxidase and generation of mitochondrial reactive oxygen species. *The FASEB Journal*.

[36] Hui W, Jinxiang Z, Heshui W, Zhuoya L, Qichang Z (2009). Bone marrow and non-bone marrow TLR4 regulates hepatic ischemia/reperfusion injury. *Biochemical and Biophysical Research Communications*.

